# PSMε controls skin commensal CD8 ^+^ T cell activation

**DOI:** 10.64898/2026.07.30.741773

**Published:** 2026-08-04

**Authors:** William S. Owens, Kevin Lenzi, Wendy Geng, Annalisa Hurd, Jessie J. Y. Liu, Caleb Staudinger, Brittany Berdy, Cameron Lian, Namrata D. Udeshi, Steven A. Carr, Christopher D. Johnston, Jonathan Livny, Y. Erin Chen

## Abstract

Upon skin colonization, the prevalent human skin commensal *S. epidermidis* can elicit a CD8 ^+^ T cell response that protects against pathogens or clears tumors. The microbial features that drive this response are undefined, limiting our ability to understand and predict commensal-immune crosstalk and to engineer potent commensal-derived immunotherapies. To uncover these microbial features, we harnessed both the natural variation in CD8 ^+^ T cell induction across primary human isolates of *Staphylococcus* and our ability to genetically manipulate these strains. Stimulatory strains exhibit increased quorum sensing activation, which turns on a unique commensal-associated gene family, called phenol-soluble modulin ε (PSMε), that is required for CD8 ^+^ T cell activation. PSMε not only acts as the immunodominant CD8 ^+^ T cell antigen but also enhances cross-presentation in an antigen-agnostic manner. Co-delivering PSMε promotes CD8 ^+^ T cell priming to an exogenous antigen via a mechanism that is independent of formyl peptide receptor and co-stimulatory receptor upregulation. Thus, we demonstrate that specific aspects of microbiome-immune crosstalk can be distilled to molecular components, which engage in previously undescribed mechanisms and can be harnessed for immunotherapy without requiring live bacterial colonization.

## Full Text Availability

The license terms selected by the author(s) for this preprint version do not permit archiving in PMC. The full text is available from the preprint server.

